# Physician density: will we ever close the gap?

**DOI:** 10.1186/s13104-023-06353-8

**Published:** 2023-05-21

**Authors:** Fabrizio Ferretti, Michele Mariani, Elena Sarti

**Affiliations:** 1 Department of Communication and Economics, University of Modena and Reggio Emilia, Viale Allegri 9, 42121 Reggio Emilia, Italy; 2 Department of Economics – Marco Biagi, University of Modena and Reggio Emilia, Via Berengario 51, 41121 Modena, Italy

**Keywords:** African countries, Convergence, Clubs, Log-t regression, Physician density

## Abstract

**Objective:**

Physician density is a crucial element of a well-functioning health system. Previous research has investigated factors affecting country-level physician supply. To date, however, no evidence has been provided about the patterns of convergence in physician density among countries. This paper thus tested club convergence in physician density in 204 countries worldwide from 1990 to 2019. A nonlinear time-varying factor model was adopted to identify potential clubs, wherein groups of countries tend to converge towards the same level of physician density. Our primary purpose was to document the potential long-lasting disparity in future global physician distribution.

**Results:**

Despite physician density increasing in all regions globally from 1990 to 2019, we found no evidence in favor of the hypothesis of global convergence. Conversely, the clustering algorithm successfully identified three main patterns (i.e., three final clubs). With few exceptions, the results indicated an uneven physician distribution between the majority of North and Sub-Saharan African countries (where physician density would remain well below the estimated threshold of at least 70% of the Universal Health Coverage Services Index) and the rest of the world. These findings support the WHO's global strategy to reverse the chronic under-investment in human resources for health.

**Supplementary Information:**

The online version contains supplementary material available at 10.1186/s13104-023-06353-8.

## Introduction

Proper health worker density is a critical component of a well-functioning health system [1, 2]. Empirical evidence indicates a strong positive association between health workforce availability, adequate universal health coverage, and several measures of population-level health outcomes [3–5]. Expanding and improving the healthcare workforce, especially in developing countries, are therefore key priorities of the global strategy on human resources for health, as outlined by the WHO [6] to implement Goal 3 (Ensure healthy lives and promote well-being for all at all ages) of the UN 2030 Agenda for Sustainable Development [7].

Given the substantial and far-reaching impact of the density of health workers on the broader socio-economic and health system development (and vice-versa), research has investigated the determinants of the supply and demand of health professionals worldwide [8–12]. So far, however, scant attention has been paid to the potential patterns of convergence in health worker density across countries and regions. This research note tested club convergence in physician density among 204 countries worldwide from 1990 to 2019. To this aim, we applied the methodology initially proposed by Phillips and Sul [13, 14] to investigate the behavior of economies in transition toward a steady state. This methodology allows the clustering of countries into groups according to their tendency to converge toward a common long-term value of any given variable of interest. Data on physician density come from the latest work of GBD 2019 Human Resources for Health Collaborators [4], which provides comprehensive and updated estimates of the number of several health worker cadres across countries. Our primary purpose is to document the potentially long-lasting disparity in the future of physician distribution between developed and developing countries.

## Methods and data

Since its introduction [15], the concept of convergence has been widely utilized to study the long-term evolution of economic variables (e.g., income per capita, healthcare expenditure, etc.) and, more recently, that of non-communicable disease risk factors, such as obesity rates and smoking prevalence [16–19]. Most of this research is usually based on the time-varying factor model developed by Phillips and Sul [13, 14]; this analytical framework tests the null hypothesis of convergence for a specific variable of interest between a given set of countries. Furthermore, if countries converge towards different values, thanks to an iterative algorithm [14], the model facilitates the endogenous identification of patterns of convergence between subsets (or 'clubs') of countries.

For our purposes, Phillips and Sul's methodology [13, 14] can be briefly outlined as follows [18]. Let us denote with *PHY*_*it*_ the density of physicians in country *i* and year *t*. Specifically, *PHY*_*it*_ can be written as the sum of a common (*g*_*it*_) and an idiosyncratic (*a*_*it*_) component (i.e., *PHY*_*it*_ = *g*_*it*_ + *a*_*it*_). This methodology focuses on the evolution of the idiosyncratic component. Thus, *PHY*_*it*_ can be transformed such that the common and idiosyncratic components are separated as follows:

1 $$PHY_{it} \, = \,\left( {\frac{{g_{it} + a_{it} }}{{\mu_{it} }}} \right)\,\mu_{t} \, = \,\delta_{it} \mu_{t}$$

In Eq. (1),* μ*_*t*_ is the common trend component across countries, and *δ*_*it*_ is a time-varying heterogeneous component, meaning that *δ*_*it*_ measures the deviation of the density of physicians in country *i* from the common path (i.e., the trend component *μ*_*t*_). Then, the next step requires the removal of the common factor. To this aim, in Eqs. (2) and (3), the relative transition parameter (*h*_it_) and its cross-sectional variation (*H*_*it*_) are defined as follows:

2 $$h_{it} \, = \,\frac{{PHY_{it} }}{{N^{ - 1} \,\sum\nolimits_{i = 1}^{N} {PHY_{it} } }}\, = \,\frac{{\delta_{it} }}{{N^{ - 1} \,\sum\nolimits_{i = 1}^{N} {\delta_{it} } }}$$

3 $$H_{it} = N^{ - 1} \sum\nolimits_{i = 1}^{N} {(h_{it} - 1)^{2} \to 0,as\,t \to \infty }$$

According to this methodology, $\underset{t\to \infty }{\mathrm{Lim}}{\delta }_{\mathit{it}}={\delta }_{i}=\delta$, for all *i* = 1, …, *N*. In other words, countries will converge towards a common value of *PHY* at some future point in time. As a result, if the *δ*_*it*_ tends to converge towards *δ,* there is evidence in favor of the convergence hypothesis (in this case, *h*_*it*_ would tend to 1 and *H*_*it*_ to 0, as time tends to infinity). The following time-series (or log-t) regression:

4 $$\log \frac{{H_{1} }}{{H_{t} }} - {2}log\left[ {log\left( t \right)} \right] \, = \, \alpha \, + \beta log\left( t \right) \, + \nu_{t} , t = \, \left[ {rT} \right] \, + { 1}, \ldots ,T$$

is the tool suggested by Phillips and Sul [13, 14] to test the null hypothesis of convergence (where *r* is usually set equal to 0.3 for annual datasets). Finally, using a robust one-sided t-test, the null hypothesis of convergence H_0_∶ δ_i_ = δ (i.e., *β* = 0) is rejected (at the 5% level) if the conventional t-statistic takes a value of lower than -1.65.

At the end of the procedure, clubs are formed based on the final density. Thus, countries that tend to converge to a common value at some future point in time are collected in the same club. As a result, the number of clubs is not decided a priori. Conversely, it is formed based on the trend of the variable of interest (N.B. it is also possible not to find any club).

Hereafter, *PHY* is measured by the number of physicians per 10,000 people in the population under study, as defined by the GBD 2019 Human Resources for Health Collaborators [4]. Data on physician density covers 204 countries and 30 years (from 1990 to 2019). Some descriptive statistics are shown in Additional file 1: Table S1. The full dataset is publicly available at: https://ghdx.healthdata.org/record/ihme-data/gbd-2019-human-resources-health-1990-2019. Finally, the econometric analyses were performed in Stata 16.1 [20], using the software package 'psecta,' developed by Du [21].

## Results

**Fig. 1:**
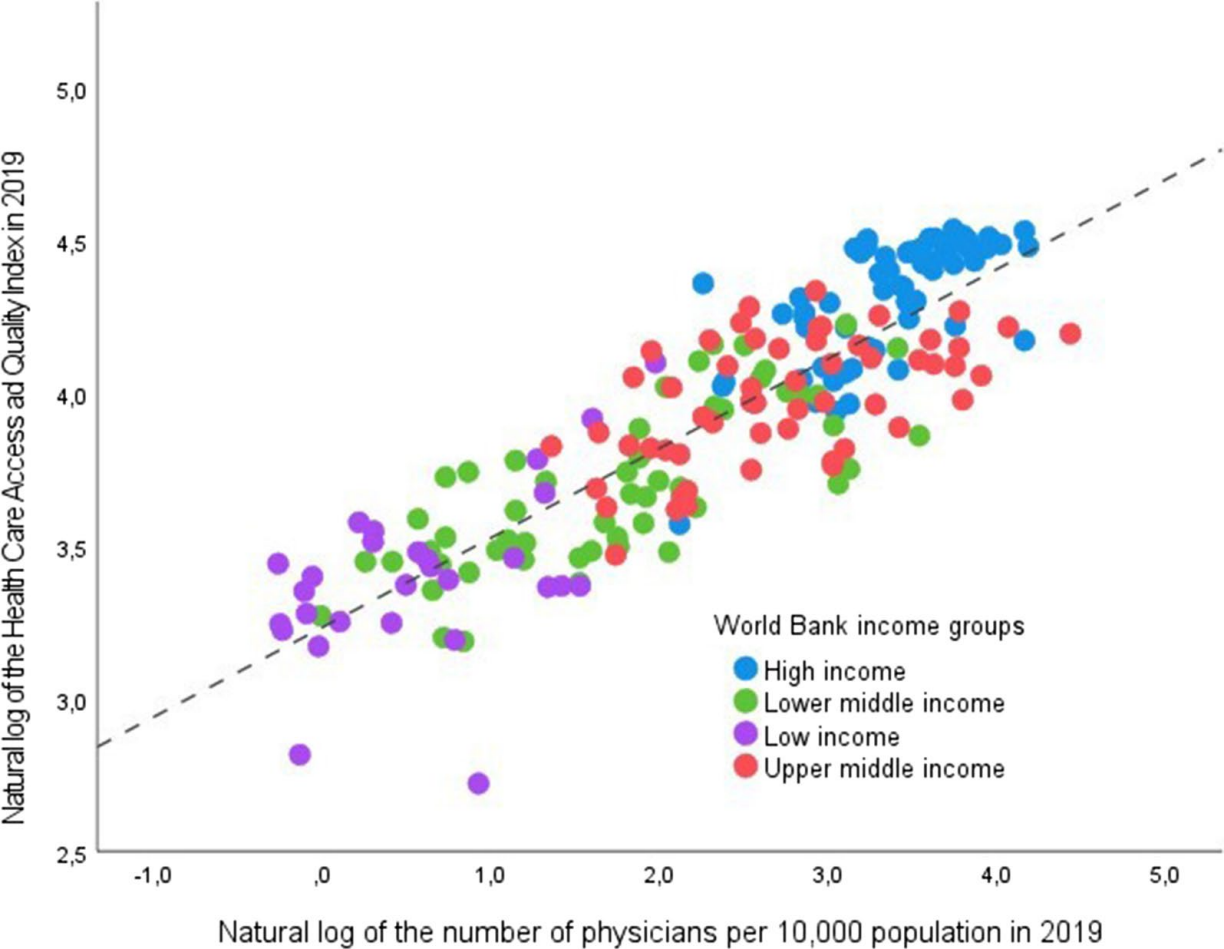
Relationship between physician density and the Health Care Access and Quality Index. Estimated regression line: ln(*HAQ*_*i*_) = 3.23 + 0.29ln(*PHY*_*i*_), *N* = 204 Adj. *R* squared = 0.75. (*PHY* is statistically significant at the 1% level)

The importance of addressing the long-term evolution of physician density is summarized in Fig. 1, where the Healthcare Access and Quality Index (*HAQ*)—a composite measure of health-system performance created by the Global Burden of Diseases Study [22]—is plotted against the number of physicians per 10,000 people in 2019 (both variables are transformed into their natural logarithms to reduce variability). Previous research has highlighted a strong correlation between the *HAQ* index and the density of the healthcare workforce [23]. The following Equation, estimated with the latest GBD data [4], provide further support to these findings:

5 $${\text{ln}}\left( {HAQ_{i} } \right) \, = {2}.{99} + \, 0.{\text{23ln}}(PHY_{i} ) \, + \, 0.{\text{11ln}}\left( {N\& M_{i} } \right)$$

(0.01) (0.02)

*t* = 12.74 *t* = 4.52*N* = 204 

$$\overline{R }$$ ^2^ = 0.78.

where *N&M* measures the number of nurses and midwives per 10,000 people [4]. Both coefficients are statistically significant at the 1% level and measure the elasticity of *HAQ* with respect to *PHY* and *N&M*. Thus, an increase in physicians' density by 10% is associated with an improvement in the accessibility and quality of healthcare services by around 2.3%. Despite the interpretation of Eq. 1) may be complicated by omitted variable bias and endogeneity, for a lower-middle-income country, such as Vietnam, this result means that doubling physician density from 7.7 to 15.4 would increase the *HAQ* by slightly less than one-fourth, from 55.6 to 68.4 (i.e., around the same value of countries like Latvia and Chile, where *HAQ* is currently 68.5 and 70.9, respectively).

**Fig. 2:**
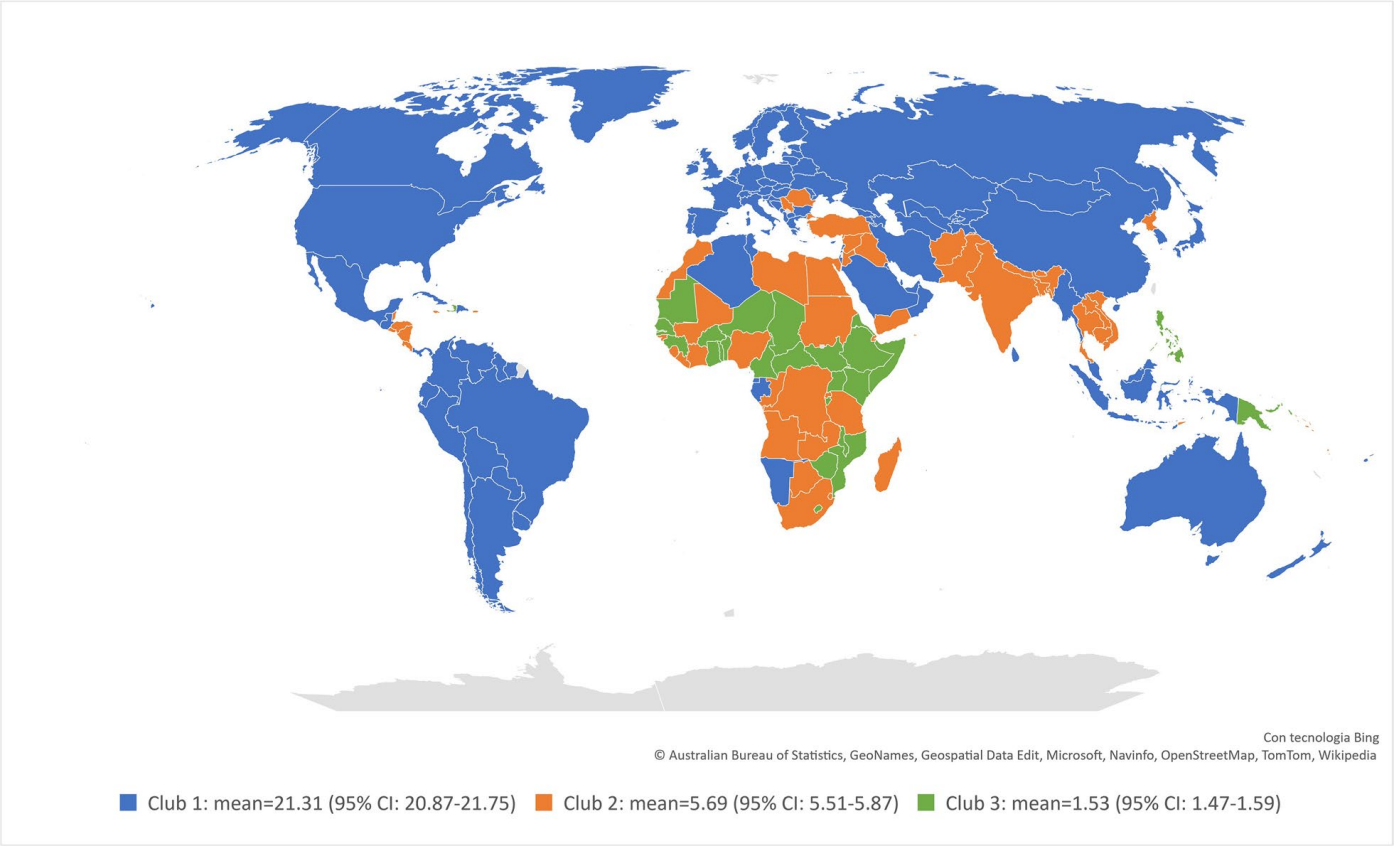
Final clubs by physician density (physicians per 10,000 people, convergence process till 2019)

Despite physician density increasing among all income groups, as reported in Additional file 1: Table S1, it does not tend to converge toward a common value. This is evidenced in Table 1, which collects the results of Phillips and Sul's [13, 14] log-t regression; the t-statistic is less than − 1.65. Thus, the null hypothesis of convergence is rejected at a 5% significance level. However, this does not preclude convergence within subsets of countries. As noted previously, the methodology enables the endogenous determination of clubs by clustering together those countries that tend to converge toward a common value of the variable under investigation. The semi-parametric clustering algorithm identified three strong clubs. The results are collected in the bottom section of Table 1 and depicted in Fig. 2 (a list of countries composing each club is provided in Additional file 1: Table S2.

**Table 1:** Results of the log-t test for convergence analysis

	Coeff	t-stat	N. of countries	N. of years
Log(t)	− 0.570	− 28.277	204	30
Club 1	0.047	0.825	110	30
Club 2	0.053	0.752	66	30
Club 3	0.028	0.448	28	30

These results may be useful for planning investments in the physician workforce, especially among developing countries, in order to anticipate future scenarios. Although the density of physicians is not the only determinant of population health outcomes, a structural shortage of physicians is a major barrier to the delivery of effective and equitable healthcare services [1, 2]. The identification of three final strong clubs highlights that, if nothing changes in the supply and allocation of physicians worldwide, the dichotomy between the vast majority of African countries and the rest of the world will tend to crystallize in the near future (as it is apparent by comparing Fig. 2 with Additional file 1: Figure S1 shows the worldwide physician distribution in 1990). This is important because the large majority of countries that fall within Clubs 2 and 3 are characterized by an average physician density well below the minimum threshold needed to achieve a universal health coverage effective score of at least 70 out of 100 [24]. Furthermore, and no less relevant for future public health issues, even in high- and upper-middle-income countries, mostly included in Club 1, physician density tends to converge, on average, towards 21 per 10,000 people, which is just above the revised threshold for achieving a goal of 80 out of 100 in the UHC effective coverage index according to the new GDB estimate [4].

A possible explanation for this result may be the relatively low responsiveness of physician density to economic growth (as measured by changes in real per capita income). According to the World Bank [25], in the Middle East & North Africa and Sub-Saharan African countries, in 2019, GDP per capita was about one and a half times that of 1990 (on average, income per capita increased by 163% and 160%, respectively). However, during the same period, the density of physicians increased by 129% in the Middle East & North Africa and only by 78% in Sub-Saharan African countries. These figures imply an elasticity of physician density with respect to income per capita well below one (129%/163% = 0.79 and 78%/160% = 0.49, respectively). In other words, better economic conditions did not turn into increases in the physician density needed to, at least partially, bridge the gap, contributing to the stagnation of these countries into a health-poverty trap [26].

In summary, we obtained three main results: (1) The composition of the final clubs highlights that the current unequal allocation is meant to be a structural feature of the future worldwide physician distribution; (2) Countries in Clubs 2 and 3 will also be characterized by an average physician density well below the minimum threshold required to respond to population health needs; and (3) Even in ‘rich’ countries (Club 1), without any action, physician shortage will become a challenging public health issue.

To the best of our knowledge, this is the first attempt to test convergence in physician density worldwide. However, previous works have investigated the long-term evolution of physician density at the country level [27–29], reporting an uneven physician distribution, but also a greater tendency to converge, driven by economic growth. Finally, we also tested club convergence for nurses and midwives, and we found that the patterns of convergence tend to replicate those observed for physicians. The results are collected in the Supplementary Information (File SI1, Tables S3, S4, and Figure S2).

### Limitations

The limitations of this study include at least five main issues. First, our main focus was on physician density. However, despite the key role played by physicians, health system performances are also determined by the density of several other health workforce cadres, such as community health workers, dentists, pharmacists, etc. Second, the data availability did not allow us to further disaggregate health cadres (for instance, to separate specialist versus generalist physicians or nurses from midwives). Third, healthcare models differ across countries. Especially in developing countries, nurses and community health workers are often the first contact point between patients and the health system, whereas physicians are usually limited to hospitals. Fourth, our data sources did not allow us to control for physician quality (i.e., cross-country variation in the levels of physician training and performance). Fifth, the paper set out to test the convergence hypothesis without investigating the determinants of physician density. Thus, further research is needed to compare the patterns of convergence among other health professionals and to analyze the main determinants of club convergence for each type of health worker.

## Supplementary Information

**Additional file 1: Table S1.** Physicians per 10,000 people. **Table S2.** Physicians. List of countries by club. **Table S3.** Nurses and midwives Results of the log-t test for convergence analysis. **Table S4.** Nurses and midwives. List of countries by club. **Figure S1.** Physician density in 1990. **Figure S2.** Final clubs by nurses and midwives’ density

## Data Availability

We used secondary data. All the data utilized in this paper are publicly available at: https://ghdx.healthdata.org/gbd-2019 for physician density and the HAQ (Health Care Access and Quality Index), available at https://data.worldbank.org/indicator/NY.GDP.PCAP.PP.KD for GDP per capita.

## Abbreviations

GBD: Global burden of diseases
GDP: Gross domestic product
HAQ: Healthcare access and quality index
UN: United Nations
WHO: World Health Organization
