# Supplementary material for: Physician density: will we ever close the gap?
Source: BMC Res Notes. 2023 May 21;16:84. doi: 10.1186/s13104-023-06353-8 (PMC10201702; doi:10.1186/s13104-023-06353-8)
Supplement: Supplementary file 1 — Additional file 1: Table S1. Physicians per 10,000 people. Table S2. Physicians. List of countries by club. Table S3. Nurses and midwives Results of the log-t test for convergence analysis. Table S4. Nurses and midwives. List of countries by club. Figure S1. Physician density in 1990. Figure S2. Final clubs by nurses and midwives’ density [file 13104_2023_6353_MOESM1_ESM.docx]

**Supplementary Information.** File S1 (.docx), including Table S1. Physicians per 10,000 people (204 countries), Table S2. Physicians. List of countries by club, Fig. S1. Physician density in 1990 (physicians per 10,000 people), Table S3. Nurses and midwives. Results of the log-t test for convergence analysis, Fig. S2. Final clubs by nurses and midwives’ density (nurses and midwives per 10,000 people), Table S4. Nurses and midwives. List of countries by club.

**Table S1.** Physicians per 10,000 people (204 countries).

|  | **1990** | | | **2019** | | |  |
| --- | --- | --- | --- | --- | --- | --- | --- |
| **Income group**^1^ | **Mean** | **SD** | **Ratio**^2^ | **Mean** | **SD** | **Ratio** | **AAGR**^3^ |
| High | 19.12 | 9.93 | 1.00 | 31.86 | 13.89 | 1.00 | 2.02 |
| Upper middle | 9.53 | 7.96 | 2.01 | 19.74 | 15.64 | 1.61 | 2.64 |
| Lower middle | 4.31 | 4.12 | 4.43 | 8.35 | 7.49 | 3.81 | 2.25 |
| Low | 1.43 | 1.11 | 13.33 | 2.24 | 1.60 | 14.23 | 1.59 |

**Note.** ^1^ World Bank country groups by income level in 2019. ^2^ Ratio of physician density in high to other income groups. ^3^ AAGR, average annual growth rate.

**Table S2.** Physicians. List of countries by club.

| **Club** | **Countries** |
| --- | --- |
| Club 1:  110 countries | Albania, Algeria, Andorra, Antigua and Barbuda, Argentina, Armenia, Australia, Austria, Azerbaijan, Bahamas, Bahrain, Barbados, Belarus, Belgium, Bermuda, Bolivia, Bosnia and Herzegovina, Brazil, Brunei Darussalam, Bulgaria, Cabo Verde, Canada, Chile, China, Colombia, Cook Islands, Croatia, Cuba, Cyprus, Czechia, Denmark, Dominican Republic, Ecuador, Equatorial Guinea, Estonia, Fiji, Finland, France, Gabon, Georgia, Germany, Greece, Greenland, Grenada, Guatemala, Guyana, Hungary, Iceland, Indonesia, Iran (Islamic Republic of), Ireland, Israel, Italy, Japan, Kazakhstan, Kuwait, Kyrgyzstan, Latvia, Lebanon, Lithuania, Luxembourg, Malaysia, Maldives, Malta, Mauritius, Mexico, Monaco, Mongolia, Montenegro, Myanmar, Namibia, Netherlands, New Zealand, North Macedonia, Norway, Oman, Palestine, Panama, Paraguay, Peru, Poland, Portugal, Qatar, Republic of Korea, Republic of Moldova, Russian Federation, Saint Kitts and Nevis, Saint Lucia, San Marino, Saudi Arabia, Singapore, Slovakia, Slovenia, Spain, Sri Lanka, Suriname, Sweden, Switzerland, Tajikistan, Trinidad and Tobago, Tunisia, Turkmenistan, Ukraine, United Arab Emirates, United Kingdom, United States Virgin Islands, United States of America, Uruguay, Uzbekistan, Venezuela. |
| Club 2:  66 countries | Afghanistan, American Samoa, Angola, Bangladesh, Belize, Bhutan, Botswana, Cambodia, Comoros, Republic of the Congo, Costa Rica, Côte d'Ivoire, Democratic People's Republic of Korea, Democratic Republic of the Congo, Djibouti, Dominica,  Egypt, El Salvador, Eswatini, Guam, Guinea-Bissau, Honduras, India, Iraq, Jamaica,  Jordan, Kiribati, Lao People's Democratic Republic, Liberia, Libya, Madagascar, Mali, Marshall Islands, Micronesia (Federated States of), Morocco, Nauru, Nepal, Nicaragua, Nigeria, Niue, Northern Mariana Islands, Pakistan, Palau, Puerto Rico,  Romania, Rwanda, Saint Vincent and the Grenadines, Serbia, Seychelles, Sierra Leone, Solomon Islands, South Africa, Sudan, Syrian Arab Republic, Taiwan (Province of China), Thailand, Timor-Leste, Tokelau, Tonga, Turkey, Tuvalu, United Republic of Tanzania, Vanuatu, Vietnam, Yemen, Zambia. |
| Club 3:  28 countries | Benin, Burkina Faso, Burundi, Cameroon, Central African Republic, Chad, Eritrea,  Ethiopia, Gambia, Ghana, Guinea, Haiti, Kenya, Lesotho, Malawi, Mauritania, Mozambique, Niger, Papua New Guinea, Philippines, Samoa, Sao Tome and Principe, Senegal, Somalia, South Sudan, Togo, Uganda, Zimbabwe. |


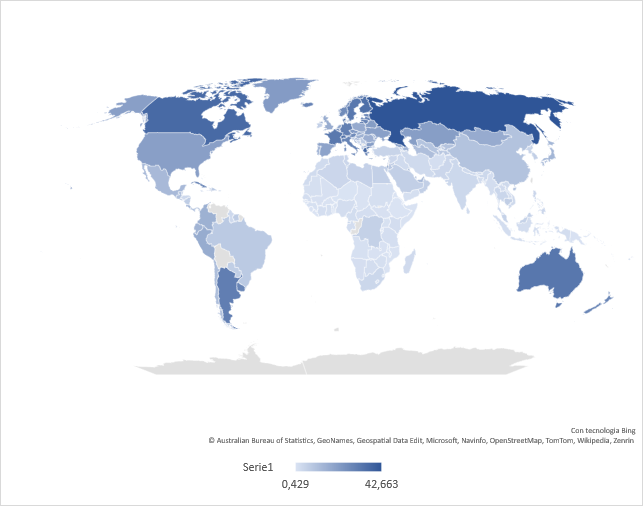


**Fig. S1.** Physician density in 1990 (physicians per 10,000 people).

**Table S3.** Nurses and midwives Results of the log-t test for convergence analysis.

|  | **Coeff.** | **t-stat** | **N. of countries** | **N. of years** |
| --- | --- | --- | --- | --- |
| Log(t) | -0.420 | -32.504 | 204 | 30 |
|  |  |  |  |  |
| Club 1 | 0.048 | 1.075 | 137 | 30 |
| Club 2 | 0.178 | 2.290 | 39 | 30 |
| Club 3 | 0.076 | 1.434 | 23 | 30 |
| Club 4 | 0.149 | 1.227 | 5 | 30 |


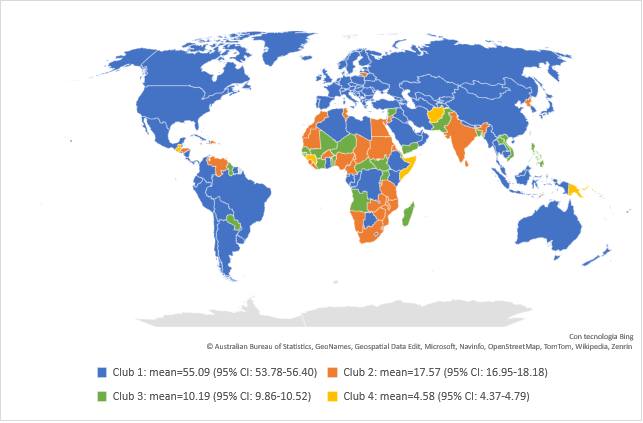


**Fig. S2.** Final clubs by nurses and midwives’ density (nurses and midwives per 10,000 people).

**Table S4.** Nurses and midwives. List of countries by club.

| **Club** | **Countries** |
| --- | --- |
| Club 1:  137 countries | Albania, Algeria, American Samoa, Andorra, Antigua and Barbuda, Argentina  Armenia, Australia, Austria, Azerbaijan, Bahamas, Bahrain, Belarus, Belgium  Belize, Bermuda, Bhutan, Bolivia, Bosnia and Herzegovina, Botswana, Brazil  Brunei Darussalam, Bulgaria, Cabo Verde, Cambodia, Canada, Chile, China,  Colombia, the Republic of the Congo, the Cook Islands, Costa Rica, Croatia, Cuba,  Cyprus, Czechia, the Democratic Republic of the Congo, Denmark, Dominica,  Ecuador, El Salvador, Equatorial Guinea, Estonia, Eswatini, Ethiopia, Fiji, Finland,  France, Gabon, Georgia, Germany, Ghana, Greece, Greenland, Grenada, Guam, Haiti, Hungary, Iceland, Indonesia, Iran (Islamic Republic of), Iraq, Ireland, Israel,  Italy, Japan, Kazakhstan, Kenya, Kiribati, Kuwait, Kyrgyzstan, Latvia, Lebanon,  Lesotho, Libya, Luxembourg, Malaysia, Maldives, Malta, Marshall Islands, Mauritius, Mexico, Micronesia (Federated States of), Monaco, Mongolia, Montenegro, Myanmar, Nauru, Nepal, Netherlands, New Zealand, Nicaragua  Niue, North Macedonia, Northern Mariana Islands, Norway, Oman, Palau,  Palestine, Panama, Peru, Poland, Portugal, Qatar, Republic of Korea, Republic of Moldova, Romania, Russian Federation, Rwanda, Saint Kitts and Nevis, Saint Vincent and the Grenadines, San Marino, Sao Tome and Principe, Saudi Arabia,  Serbia, Seychelles, Singapore, Slovakia, Slovenia, Solomon Islands, Spain, Suriname, Sweden, Switzerland, Taiwan (Province of China), Tajikistan, Thailand,  Tokelau, Turkey, Turkmenistan, Ukraine, United Arab Emirates, United Kingdom,  United States Virgin Islands, United States of America, Uruguay, Uzbekistan. |
| Club 2:  39 countries | Barbados, Burkina Faso, Burundi, Cameroon, Chad, Comoros, Democratic People's Republic of Korea, Dominican Republic, Egypt, Eritrea, Gambia, Honduras, India, Jamaica, Jordan, Liberia, Lithuania, Malawi, Mauritania, Morocco, Mozambique,  Namibia, Nigeria, Puerto Rico, Saint Lucia, Sierra Leone, South Africa, Sri Lanka,  Sudan, Timor-Leste, Tonga, Trinidad and Tobago, Tunisia, Tuvalu, United Republic of Tanzania, Vanuatu, Venezuela, Zambia, Zimbabwe. |
| Club 3:  23 countries | Angola, Bangladesh, Benin, Central African Republic, Côte d'Ivoire, Djibouti, Guinea-Bissau, Guyana, Lao People's Democratic Republic, Madagascar, Mali, Niger, Pakistan, Paraguay, Philippines, Samoa, Senegal, South Sudan, Syrian Arab Republic, Togo, Uganda, Vietnam, Yemen. |
| Club 4:  5 countries | Afghanistan, Guatemala, Guinea, Papua New Guinea, Somalia. |
